# Model‐based and model‐free mechanisms in methamphetamine use disorder

**DOI:** 10.1111/adb.13356

**Published:** 2023-12-21

**Authors:** Alex H. Robinson, Justin Mahlberg, Trevor T.‐J. Chong, Antonio Verdejo‐Garcia

**Affiliations:** ^1^ Turner Institute for Brain and Mental Health School of Psychological Sciences Monash University Melbourne Australia

**Keywords:** decision‐making, dependence, longitudinal, methamphetamine, model‐based, model‐free

## Abstract

People with methamphetamine use disorder (MUD) struggle to shift their behaviour from methamphetamine‐orientated habits to goal‐oriented choices. The model‐based/model‐free framework is well suited to understand this difficulty by unpacking the computational mechanisms that support experienced‐based (model‐free) and goal‐directed (model‐based) choices. We aimed to examine whether 1) participants with MUD differed from controls on behavioural proxies and/or computational mechanisms of model‐based/model‐free choices; 2) model‐based/model‐free decision‐making correlated with MUD symptoms; and 3) model‐based/model‐free deficits improved over six weeks in the group with MUD. Participants with MUD and controls with similar age, IQ and socioeconomic status completed the Two‐Step Task at treatment commencement (MUD *n* = 30, Controls *n* = 31) and six weeks later (MUD *n* = 23, Controls *n* = 26). We examined behavioural proxies of model‐based/model‐free decisions using mixed logistic regression, and their underlying mechanisms using computational modelling. At a behavioural level, participants with MUD were more likely to switch their choices following rewarded actions, although this pattern improved at follow up. At a computational level, groups were similar in their use of model‐based mechanisms, but participants with MUD were less likely to apply model‐free mechanisms and less likely to repeat rewarded actions. We did not find evidence that individual differences in model‐based or model‐free parameters were associated with greater severity of methamphetamine dependence, nor did we find that group differences in computational parameters changed between baseline and follow‐up assessment. Decision‐making challenges in people with MUD are likely related to difficulties in pursuing choices previously associated with positive outcomes.

## Graphical abstract

People with methamphetamine use disorder have difficulties repeating rewarding actions, compared with well‐matched controls. However, this difficulty appears to improve after six weeks.

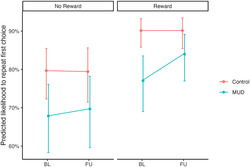

## 1 INTRODUCTION

Methamphetamine use disorder (MUD) is a global health challenge^1^ that continues to increase in prevalence,^2^, ^3^, ^4^ non‐fatal harms,^5^, ^6^ and mortality.^2^, ^7^, ^8^ People with MUD struggle to shift their behaviour from entrenched methamphetamine‐orientated actions to actions that align with long‐term goals.^9^, ^10^ Computational decision‐making frameworks are well suited to unpack this conflict, as they allow the identification of specific mechanisms that drive maladaptive decisions.^11^, ^12^ In particular, the model‐based/model‐free framework of decision‐making^13^ investigates whether people make decisions by considering future goals (i.e., recovery‐orientated actions) or simply repeating previously rewarding actions (i.e., methamphetamine use).

The model‐based/model‐free framework^13^ theorises that there are two decision‐making strategies. The first is the model‐based system, which learns 1) how environmental ‘states’ link to one another in a cognitive map and 2) the value of different actions in each state.^14^ This system allows prospective consideration of how specific actions lead to particular outcomes, and thus enables behavioural adaptations when outcome values change (i.e., shift away from methamphetamine use when it starts to harm).^11^ Conversely, in the model‐free system decisions are informed only by the value of past actions, without taking into account how aspects of the environmental may prospectively link to one another.^14^ As such, a model‐free approach does not allow a nuanced understanding of how a decision‐making environment is structured but instead enables quick and computationally efficient decisions.^13^, ^15^, ^16^

Only two studies have investigated model‐based/model‐free decision‐making in MUD. The first investigated participants in residential rehabilitation,^17^ who had been abstinent for up to one year. The main model‐based/model‐free outcome measure was a weighting parameter between the two systems (ω parameter). Results showed that participants with MUD were biased towards model‐free over model‐based decision‐making, relative to controls. However, a limitation of this approach is that it could not ascertain the integrity of each model‐free and model‐based system. Furthermore, many control participants in this study were drawn from a different cultural demographic, relative to the group with MUD. The second study was a rodent model,^18^ wherein rats were tested prior to methamphetamine administration, and again after five days of abstinence. The main outcomes were two individual parameters of model‐based (*β*_*MB*_) and model‐free learning (*β*_*MB*_), which allowed an assessment of the overall strength of these systems, rather than simply the ratio of each system being used compared with one another. Similar to the human study, methamphetamine use was associated with reduced model‐based decision‐making. However, methamphetamine‐exposed rats also had deficits in model‐free decision‐making, driven by difficulties in switching behaviour after unrewarded trials. Furthermore, greater model‐free deficits prior to methamphetamine administration led to more frequent methamphetamine use. Together, these two studies highlight the importance of quantifying the strength of both model‐based and model‐free mechanisms.^18^

Two other key aspects of model‐based/model‐free decision‐making remain unexplored in MUD. The first is whether model‐based/model‐free outcomes correlate with patterns of methamphetamine use and MUD symptomology. For example, functional imaging studies have shown that weaker model‐based and stronger model‐free brain activations during decision‐making can predict increased severity of alcohol dependence in young men.^19^ The second consideration is whether people with MUD can show improvement in model‐based/model‐free deficits across recovery. Understanding longitudinal changes (or lack of thereof) may allow a finer understanding of the link between decision‐making and clinical prognosis.^20^

Here, we examined the decision‐making profile of people with MUD by comparing their model‐based and model‐free behaviour and computational parameters with a group of well‐matched controls. We used an exploratory approach to investigate three specific aims: 1) to identify if there were differences in model‐based/model‐free decision‐making between people with MUD who had recently engaged with treatment and controls with similar sociodemographic characteristics; 2) to identify if specific mechanisms of model‐based/model‐free decision‐making correlated with patterns of methamphetamine use; and 3) to examine if people with MUD exhibit changes in model‐based/model‐free decision‐making across the early months of recovery.

## 2 METHODS

### 2.1 Design and aims

We compared model‐based/model‐free decision‐making in participants with MUD and controls at two timepoints: upon first contact with the research team during treatment and six weeks afterwards. We chose follow‐up at six weeks as the first months of recovery in MUD are associated with the greatest risk of relapse.^21^, ^22^

### 2.2 Participants

Thirty participants with MUD (*M*_*Age*_ = 34.07, *SD* = 9.42; 24 males) were compared with 31 controls without current substance use or a history of substance use disorders (*M*_*Age*_ = 31.36, *SD* = 8.53; 24 males). Participants with MUD were recruited from public and private drug and alcohol treatment organisations across Melbourne, Australia. Participants with MUD were eligible if they had 1) recently engaged in treatment; 2) reported methamphetamine as their primary drug of concern; and 3) used methamphetamines at least weekly in the month before engaging in treatment. Table 1 summarises methamphetamine use patterns, severity of dependence and treatment modality. We did not exclude participants who had concurrent use of other substances (see Table S1). Participants with MUD were required to be abstinent from methamphetamine for at least 48 h and no more than six months based on self‐report at study intake.

**TABLE 1:** Descriptive statistics of sociodemographic group comparisons and methamphetamine use characteristics in people with MUD and drug‐free controls at baseline.

Demographics	PwMUD	HC	Test statistic	Bayes factors (*BF* ^10^)
Sex (F/M)	6/24	7/24	*χ* ^2^ = 0.06, *p* = 0.81	0.26
Age	34.07 (9.42)	31.36 (8.53)	*U =* 389, *p* = 0.28	0.48
Years of education	13.82 (2.44)	14.48 (1.81)	*U =* 519, *p* = 0.44	0.41
Verbal IQ	108.81 (6.85)	110.33 (5.24)	*U* = 520, *p* = 0.43	0.33
Sociodem. status	6.60 (2.63)	7.23 (2.27)	*U* = 507, *p* = 0.40	0.37

Meth use (MUD only)	*M* (or *N*)	*SD* (or %)	Range	
Severity of dependence (SDS)	9.13	3.97	[2–15]	
Daily dose (grams)	0.52	0.74	[0.10–4.00]	
Frequency (days/month)	17.7	10.36	[ 3 –31]	
Duration (years)	9.66	6.40	[1–29]	
Last Use (days)	27.80	31.91	[3–151]	
*Form of treatment*				
Inpatient rehab	11	36.67%	*‐*	
Outpatient counselling	12	40.00%	‐	
Multiple	6	20.00%	*‐*	
Yet to commence	1	3.33%	‐	
*Route of administration*				
Smoking	23	76.67%	‐	
Injecting	7	23.33%	‐	

*Note*: Three PwMUD also reported HIV + status. SDS scores can range between 0 and 15; with those above 4 indicating likely MUD.^29^

Controls were recruited using online advertisements and selected to have similar sociodemographic characteristics to the group with MUD (i.e., age, gender, education, socioeconomic status, Table 1). Nonetheless, controls reported lower levels of depression^23^ [*M* (*SD*) = 11.29 (9.32)], compared with participants with MUD [*M* (*SD*) = 18.90 (9.47), *U* = 228, *p* < 0.001, *BF*_*10*_ = 19.06]. Controls were included if they had used illicit substances less than 10 times in the past five years and did not have a history of substance use disorders (self‐report). We also cross‐validated and confirmed self‐report using hair toxicology including methamphetamine, other stimulants, opioids, cannabis and cannabinoids. Exclusion criteria for both groups included a history of bipolar, schizophrenia or acquired brain injury (self‐reported), and IQ estimates that could impact the understanding of the tasks (score <80 on the National Adult Reading Test [NART]).^24^ Baseline data from approximately half this sample has been reported for another decision‐making task.^25^

Twenty‐three (~76%) participants with MUD returned for follow‐up. Seven did not return: two could not be contacted, two reported scheduling difficulties, two entered inpatient treatment with restricted access and one was incarcerated. Participants with MUD who returned to follow‐up reported a reduction in their frequency of methamphetamine use by an average of 12.73 (*SD* = 11.93) days (comparing the past four weeks of methamphetamine use at follow‐up with the past four weeks in active use prior to treatment), and only two participants had not reduced their methamphetamine use. In the control group, follow‐up data was available from 26 participants (~84%). Four participants could not attend the follow‐up session (two reported scheduling difficulties and two were unavailable because of COVID‐19 restrictions). Furthermore, one control reported using MDMA between assessments, thus their follow‐up data was excluded. Table S2 reports demographics for participants who completed both sessions.

### 2.3 Procedure

The Eastern Health Human Research Ethics Committee approved the study (E52/1213). Assessments occurred at treatment facilities or nearby community libraries. Participants were initially screened for eligibility and then underwent a standardised assessment session (1.5 h). Participants returned for the follow‐up session, six weeks later. Upon completion, participants were reimbursed with a $40 (AUD) gift‐card and a report summarising their decision‐making performance.

### 2.4 Measures

#### 2.4.1 Sociodemographic characteristics

Verbal IQ was assessed by the NART. Sociodemographic status was estimated by participant's postcodes and the Australian Bureau of Statistics, Socio‐Economic Indexes for Areas tool.^26^

#### 2.4.2 Methamphetamine use patterns and secondary drug use

Frequency of methamphetamine use in the four weeks before treatment was measured by the Timeline Follow Back Interview (TLFB).^27^ Severity of methamphetamine dependence was measured using the Severity of Dependence Scale (SDS).^28^, ^29^ Lifetime patterns of substance use (i.e., average dose, years of methamphetamine use, preferred form and secondary substance use) were estimated by the Interview for Research on Addictive Behaviours (IRAB).^30^

#### 2.4.3 Biological measure of substance use

We collected hair samples at follow‐up from all participants to confirm accurate self‐report from participants with MUD and to confirm that controls were not using any illicit substances. These samples were analysed using gas chromatography–mass spectrometry.^31^ There was a strong correlation between self‐reported methamphetamine use on the Timeline Follow Back and methamphetamine concentrations in hair (ng/mg; *rho* = 0.74, *p* < 0.001).

#### 2.4.4 Two‐step decision‐making task

**FIGURE 1:**
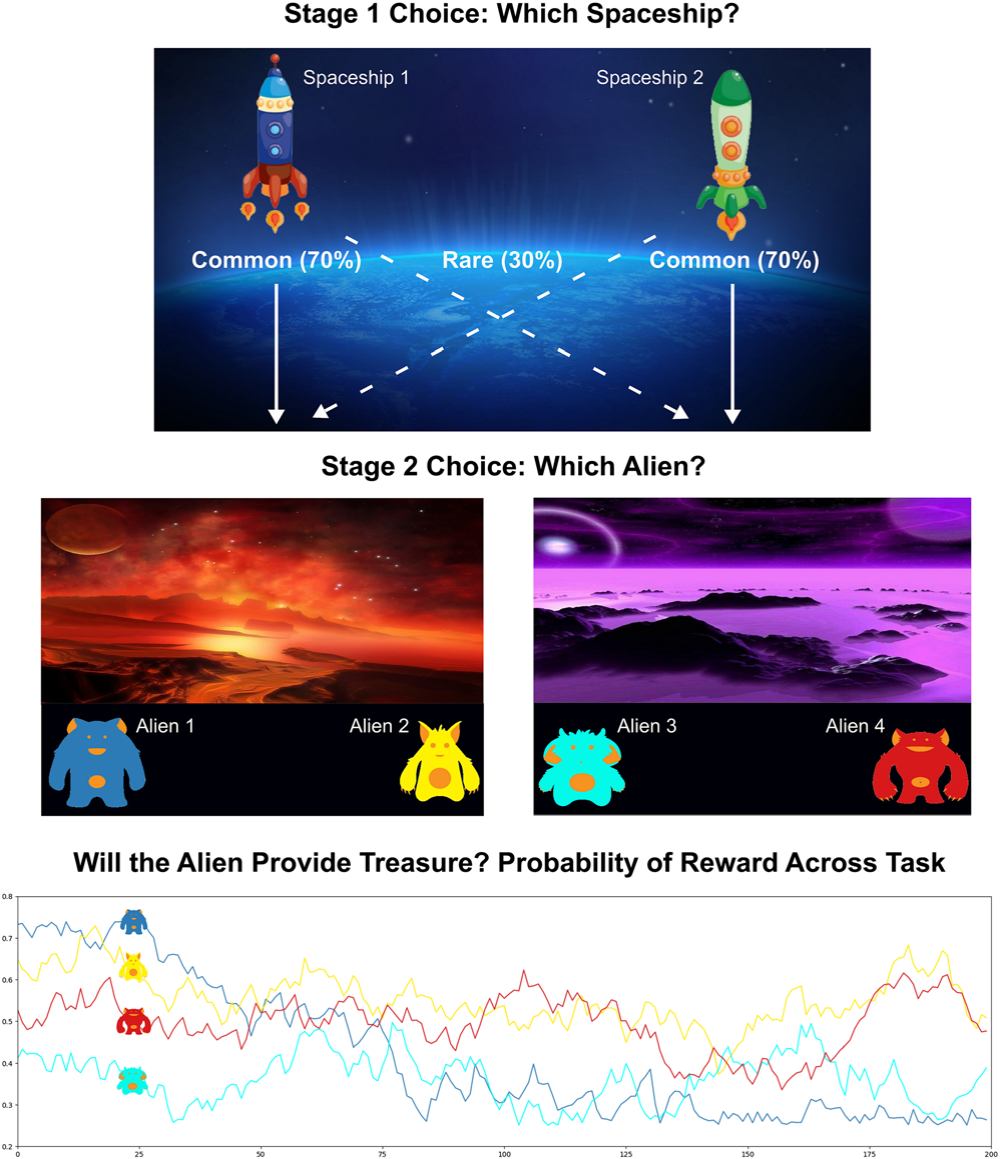
Diagram of Two‐Step Task. *Note*: At stage 1, participants choose between two spaceships. The blue spaceship has a 70% chance to take them to the red planet (common transition) and a 30% chance to take them to the purple planet (rare transition). The opposite probabilities apply for the green spaceship. Upon arriving on either planet, the participant then must choose between two different aliens, which have different probabilities of providing reward across time

This computer task requires participants to make two consecutive choices to obtain fictive in‐game rewards (see Figure 1).^13^ At the first choice, participants decide between two spaceships (blue or green), each with a fixed 70/30% probability of taking them to the red/purple planet, and vice‐versa. At the second choice, participants choose between two aliens on the planet they land on, with each alien having different likelihoods of rewarding the participant with one alien treasure. The likelihood of each aliens' reward changes independently over time, via four pre‐determined Gaussian walks. These walks were counterbalanced within the MUD participants at baseline and remained the same for each participant at follow‐up. The control group received counterbalancing that matched the group with MUD. Before commencing the task, participants completed a short tutorial that included instructions and 50 practice trials (10 response training trials, where they learned how to select aliens; 20 second stage alien choice practice trials, where they learnt how to select between two aliens with likelihoods of providing reward; and 20 full practice trials). They then completed 200 trials. It is important to note that this version of the Two‐Step Task – the most common version used in the addiction literature – does not necessarily reward a model‐based strategy more than a model‐free strategy.^32^ As such, performance likely reflects participants' ‘default’ decision‐making style. We provide test–retest reliability data for our main outcomes in the Supplementary Materials.

### 2.5 Statistical analyses

#### 2.5.1 Overview

For hypothesis testing, we used a frequentist approach with *α* = 0.05, incorporating Bayesian statistics where possible. For Bayesian statistics, the alternate hypothesis was that participants with MUD would differ from controls (two tailed; *BF* > 3 implies evidence in favour of the alternate hypothesis; *BF* < 1/3 implies evidence in favour of the null).^33^ Sample size was based on previous addiction studies of the Two‐Step Task.^17^, ^34^, ^35^

#### 2.5.2 Aim 1: baseline differences in model‐based/model‐free decision‐making between people with MUD and controls

##### Data cleaning

We applied previously established criteria to identify suboptimal engagement in the Two‐Step Task.^36^ This included missing >10% of trials, responding with the same key on more than 95% of trials across both stages and having reaction times that were more than two standard deviations faster than other participants. As such, we excluded one participant with MUD who omitted responses on 19.5% of trials at the initial assessment.

##### Overt behaviour

Two‐Step Task choices were analysed with a mixed generalised logistic regression.^13^, ^17^, ^36^ The outcome variable was ‘Stay’ (whether participants repeated their first stage choice, [0, 1]). Fixed predictors included Previous Win (whether participants were rewarded on the previous trial, [−1, 1]); Previous Transition (whether the transition to the planet on the last trial was rare or common, [−1, 1]); the interaction between Previous Win * Previous Transition; Group (MUD or control); and the interactions between Group and Previous Win/Previous Transition/Previous Win * Previous Transition. Random effects included Participant.

A significant effect of Group indicates one group was repeating first stage choices more than another. A significant effect of Previous Win indicates model‐free behaviour (e.g., a difference towards stay probability in rewarded trials, relative to non‐rewarded trials) as participants are repeating their first choice because they were rewarded, regardless of transition type. Therefore, a Group * Previous Win interaction implies group differences in this behavioural proxy of model‐free decision‐making. A significant Previous Win * Previous Transition interaction implies a behavioural pattern consistent with model‐based decision‐making (i.e., participants are considering both reward and transition type). Therefore, a Group * Previous Win * Previous Transition interaction suggests group differences in this behavioural proxy of model‐based decision‐making.

We ran this logistic regression using the *glmer* function from the *lme4* package^37^ (Version 1.1‐23), a logit link, and ‘bobyqa’ optimiser in *R*.^38^ Post‐hoc tests for significant ‘Group’ interactions involved calculating estimated marginal means using the *emmeans* package^39^ (Version 1.7.5), and applying a Tukey adjustment for multiple comparisons.

##### Computational mechanisms of model‐based/model‐free decision‐making

To understand the underlying mechanisms driving model‐based/model‐free behaviour, we implemented a well‐validated computational model of model‐based/model‐free behaviour.^40^ This approach has improved parameter estimation and group level differences in clinical populations, compared with previous approaches.^40^ In this model, every action at stage 1 (blue or green spaceship) and stage 2 (aliens on each planet) has a value, *Q*. Higher *Q*s reflect higher action values. On each trial, the *Q* values of these actions are updated based on feedback received at each stage (Equations (1 and (2).

(1) $$\;\text{Spaceship Basic Value}.\;Q_{t+1}^{\text{stage}\;1}\left(c_{1}\right)=\left(1–\alpha \right)*Q_{t}^{\text{stage}\;1}\left(c_{1,t}\right)+\left(r_{t}*0\right)$$

(2) $$\text{Alien Value}.\;Q_{t+1}^{\text{stage}\;2}\left(s_{t},c_{2,t}\right)=\left(1–\alpha \right)*Q_{t}^{\text{stage}\;2}\left(s_{t},c_{2,t}\right)+r_{t}$$

In these equations, *t* is the trial number (1–200), *c*_*1*_ */c*_*2*_ is the first/second stage choice made on that trial (left or right selection of spaceship at stage 1 or alien at stage 2), *s* is the state visited at the second stage (red or purple planet) and *r* is the reward outcome at that stage. An individual's learning rate at each stage is determined by the alpha parameter (α), where smaller values imply faster learning from feedback. At stage 1, participants do not receive reward (they simply move to the red or purple planet; *r =* 0). At stage 2, the reward, *r*, is either 1 (reward) or 0 (no reward), which then updates the value of each option.

Next, the *Q* values are used to update the value of model‐based choices (*Q*_*MB*_) and model‐free choices (*Q*_*MF*_) at the first stage. For the model‐based values, we take the maximum value of the two second stage choices from the common transition as the value for each first stage choice (Equation (3):

(3) $$\;\mathrm{MB}\;\text{First Stage Value}.\;Q_{\mathrm{MB}}\left(c_{1}\right)=\max Q_{t}^{\text{stage}\;2}\left(s,c_{2}\right)$$

In comparison, the model‐free values are updated by applying the reinforcement learning that occurred at the second stage (i.e., Equation (2) to the spaceship that was chosen on that trial. This allows the first stage choices to acquire value independent of the transition likelihoods (Equation (4).

(4) $$\;\mathrm{MF}\;\text{First Stage Value}.\;Q_{\mathrm{MF}}\left(c_{1,t+1}\right)=\left(1-\alpha \right)*Q_{t}^{\text{stage}\;2}\left(s_{t},c_{2,t}\right)+r_{t}$$

First and second stage choice probabilities are then computed by softmax functions. At stage 1, the respective values of model‐based and model‐free choices are linked to parameters that measure their individual weighting on each choice (*β*_*MB*_ and *β*_*MF*_; Equation (5). *I* is also included as a binary parameter to indicate whether each first stage choice is a repetition of the previous first stage choice. *I* interacts with *β*_*rep*_ — a parameter that identifies how biased participants are to repeat first stage choices, regardless of value.

(5) $$\text{Stage}\;1\;\text{Softmax.}P\left(c_{1,t}=c\right)\propto \exp(\beta_{\mathrm{MB}}*Q_{\mathrm{MB}}\left(c,t\right)+\beta_{\mathrm{MF}}*Q_{\mathrm{MF}}\left(c,t\right)+\;\beta_{\mathrm{rep}}I\left(c=c_{1,t-1}\right))$$

Next, the stage 2 softmax computes the probabilities for each second stage choice at the visited planet. This is based on each alien's value, and *β*_*consistency*_ measures how consistently the participant chooses the most rewarding *Q* ^*stage 2*^ values (Equation (6).

(6) $$\text{Stage}\;2\;\text{Softmax}.\;P\left(c_{2,t}=c\right)\propto \exp\left(\beta_{\text{consistency}}*Q_{t}^{\text{Stage}\;2}\left(s_{t,c}\right)\right)$$

Finally, at the end of each trial, the *Q* value of all unchosen spaceships and aliens is weighted by a ‘forgetting’ or decay function, which is the same parameter as the learning rate (1 − α). This means that the participant learns and forgets information at the same rate (Equations 7 and 8):

(7) $$\text{Forgetting Unchosen Spaceship}.\;Q_{t+1}^{\text{stage}\;1}\left(c_{\text{unchosen}}\right)=\left(1-\alpha \right)*Q_{t}^{\text{stage}\;1}\left(c_{\text{unchosen}}\right)$$

(8) $$\text{Forgetting Unchosen Aliens}.\;Q_{t+1}^{\text{stage}\;2}\left(c_{\text{unchosen}}\right)=\left(1-\alpha \right)*Q_{t}^{\text{stage}\;2}\left(c_{\text{unchosen}}\right)$$

In summary, this model provides five key parameters of computational decision‐making mechanisms: *α*, *β*_*consistency*_, *β*_*MB*,_ *β*_*MF*,_ and *β*_*rep*_ (Table 2). We fit the parameters for each session jointly, which allowed us to determine maximum a posteriori estimates for parameters in each session by using empirical priors fit across both sessions.^41^ This has been shown to be a superior approach to estimating reliability for longitudinal behavioural data.^41^ We fit the model 500 times using 10 different starting values, and selected the best‐fitting parameter values with negative log‐likelihood computed by the *emfit* package implemented in Matlab R2020b.

**TABLE 2:** Description of parameters in computational model.

Parameter name	Bounds	Description	Direction
Alpha (α)	α ∈ [0, 1]	Both learning and forgetting/decay rate of value.	Lower values reflect faster learning from reinforcement and faster forgetting of value in unselected options.
Beta MB (*β*_*MB*_)	*β*_*MB*_ ∈ [0, ∞)	Weight of model‐based decision‐making at the first stage.	Greater values reflect a greater use of model‐based strategy.
Beta MF (*β*_*MF*_)	*β*_*MF*_ ∈ [0, ∞)	Weight of model‐free decision‐making at the first stage.	Greater values reflect a greater use of model‐free strategy.
Beta Rep (*β*_*rep*_)	*βrep* ∈ (−∞, ∞)	Degree of bias to repeat first choice, disregarding value.	Positive/negative values mean a bias towards/against repetition.
Beta consistency (*β*_*consistency*_)	*βstage 2* ∈ [0, ∞)	Consistency in selecting alien with the highest value.	Greater values reflect more consistency with alien value.

To ensure that our parameters were reliable, we generated 100 synthetic datasets based on the parameter estimates for each participant, and assessing the correlation between the recovered parameters and the original parameter values. Running this recovery analysis revealed excellent recoverability for all parameters in both session 1 (*α* = 0.99, *β*_*consistency*_ = 0.99, *β*_*MB*_ = 0.82, *β*_*MF*_ = 0.98, *β*_*rep*_ > 0.99, all *p* < 0.001) and session 2 (*α* = 0.98, *β*_*consistency*_ = 0.94, *β*_*MB*_ = 0.93, *β*_*MF*_ = 0.93, *β*_*rep*_ = 0.97, all *p* < 0.001; Figure S1).

We used an exponential transform to ensure model‐based and model‐free parameter values were positive, and alpha was inverse log‐transformed to constrain it between 0 and 1 (see Table 2). We performed between‐group comparisons of each of the five free parameters using Bayesian Mann–Whitney U *t*‐tests (as the parameter distributions violated the assumption of normality). Bayesian statistics were conducted in JASP Version 0.16.2^42^ using default priors.

We ensured that each participant's choices were not random by comparing each participant's model fit with that of a control model, in which choices at each stage were random. We confirmed that the main model described above had a better fit than the chance model for the majority participants using a likelihood ratio test, which showed all but two MUD participants had a better model fit than the chance model (*p* < 0.05). We excluded those participants from analyses involving the model parameters, and the outcomes of the overt behavioural analyses are the same regardless of whether these participants are included.

#### 2.5.3 Aim 2: relationship between computational mechanisms of model‐based/model‐free decisions and methamphetamine use patterns

We examined the relationship between methamphetamine use patterns and the model‐based/model‐free computational parameters in the initial assessment. We first calculated Spearman's rho between all variables (see Figure S2 for all correlations), followed by multiple regressions for each methamphetamine use pattern variable with the computational mechanisms as predictors. We included days since last use as a covariate for all regressions. We removed one participant from these analyses who had an extreme number of days since last use (151 days) and another participant who had an extreme average dosage score for the corresponding analysis (Dosage = 4 g.). All outcome variables were mean‐centred regressions. There were moderate‐to‐strong relationships between the computational parameters; however, variance inflation factor estimates for each parameter within each fitted model suggested that there was little risk of multicollinearity impacting the regression coefficients (Table S3).

#### 2.5.4 Aim 3: longitudinal analyses of changes in model‐based/model‐free decision‐making

We examined how the behavioural proxies and mechanistic indicators of model‐based/model‐free decision‐making changed over six weeks. We excluded follow‐up data from one control participant who responded with a left action on 96.75% of choices, and the MUD participant with the very abnormal *β*_*MF*_ value at baseline.

To investigate changes in the behavioural proxies of model‐based and model‐free decision‐making, we repeated the mixed logistic regression from the initial assessment and added a fixed effect of Time, together with its interactions with Group, Previous Win and Previous Win * Previous Transition. We included Time and Participant as random effects. An interaction of Time * Group * Previous Win would indicate groups differed in how their behavioural proxy of model‐free decision‐making changed over time. An interaction of Time * Group * Previous Win * Previous Transition would indicate groups differed in how their behavioural proxy of model‐based decision‐making changed over time.

To investigate any changes in computational mechanisms of model‐based/model‐free decision‐making, we conducted 2 (Group) by 2 (Time) ANOVAs for each computational parameter. The Bayesian and frequentist analyses were conducted in JASP using default priors and estimating Bayes Factors across matched models.^43^

## 3 RESULTS

### 3.1 Aim 1: baseline differences between groups

#### 3.1.1 Overt behaviour

**FIGURE 2:**
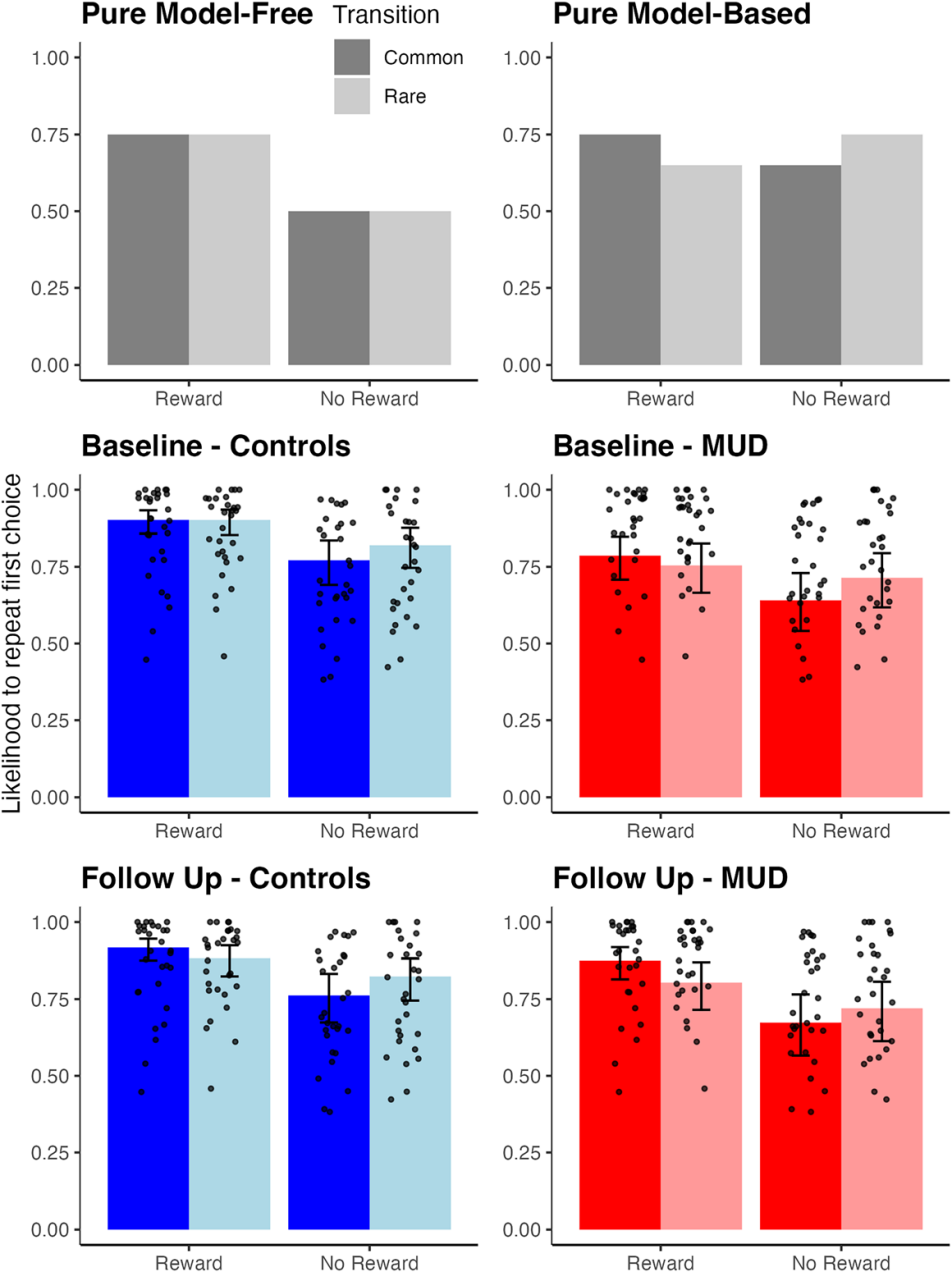
Comparison of overall behaviour at baseline and follow‐up, compared with pure model‐free and model‐based behaviour. *Note*: These figures present the observed likelihood of participants to repeat an action after reward (common, then rare transition) and non‐reward (common, then rare transition). The first row presents hypothetical examples of someone who is acting in a manner that encapsulates pure model‐free or model‐based overt behaviour. The second and third rows present observed data from the baseline and follow‐up sessions

**FIGURE 3:**
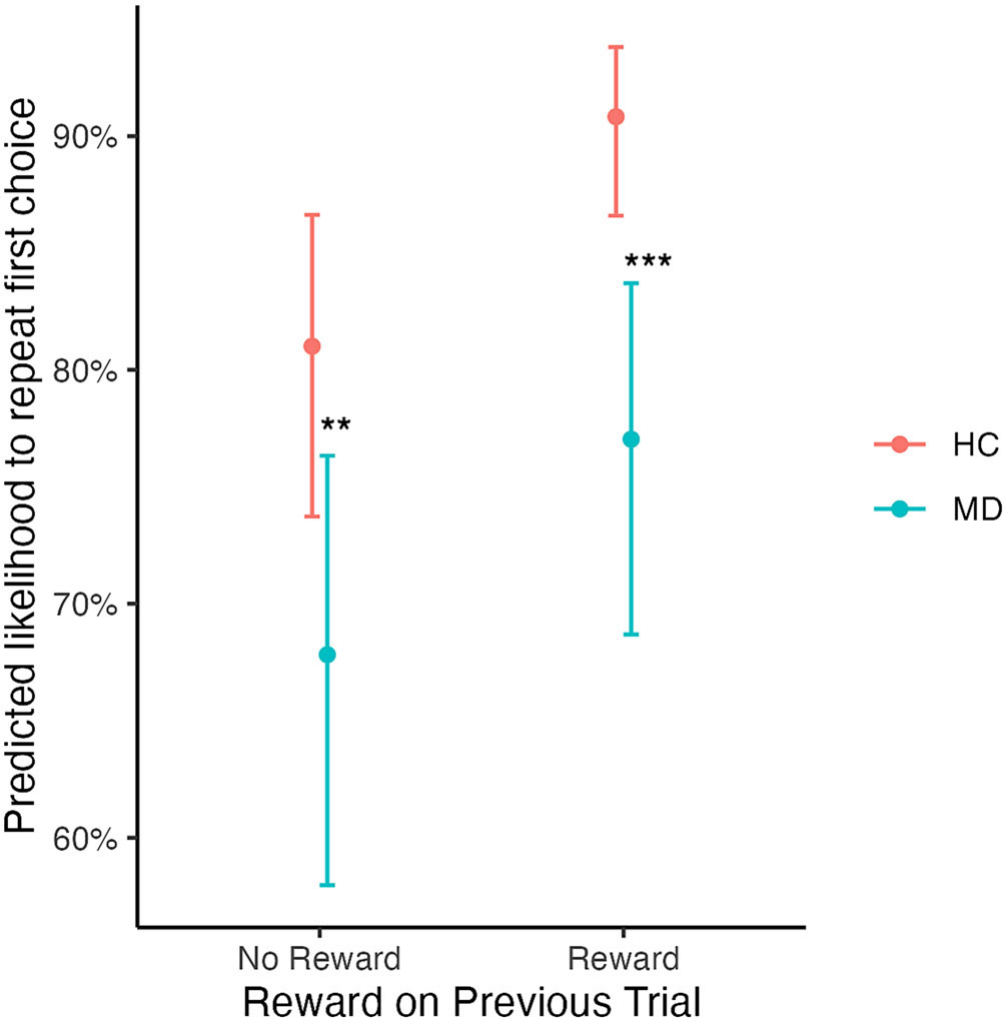
Predicted likelihood to repeat choice after different feedback and transitions. *Note*: Data points indicate the model estimated marginal mean probabilities, and the error bars indicate the 95% confidence intervals. The x axis plots whether a reward was received on the previous trial. Control = matched control participants; MUD = people with methamphetamine use disorder. **p < 0.01; ***p < 0.001

Figure 2 presents a visual comparison of participants' behaviour, compared with pure model‐free and model‐based behaviour. Overall, participants with MUD were less likely to repeat actions (Group; *OR* = 0.41, *CI* [0.25, 0.69], *p* = 0.001). Participants with MUD were significantly less likely to use feedback from the previous trial to guide their current decision — the behavioural proxy of model‐free decision‐making (Group * Previous Win; *OR =* 0.83, *95% CI* [0.75, 0.92], *p* < 0.001; Table 3). As Figure 3 illustrates, the group with MUD had a smaller effect for repeating rewarded actions, relative to non‐rewarded actions, compared with the controls. Visually, this is seen in overlap between the confidence intervals of likelihood to repeat rewarded (*OR* = 3.36, *95% CI* [2.19, 5.14]) and non‐rewarded actions (*OR* = 2.11, *95% CI* [1.38, 3.23] in the group with MUD but not the control group (rewarded *OR* = 9.88, *95% CI* [6.46, 15.12] and unrewarded *OR* = 4.27, *95% CI* [2.81, 6.48]). Post‐hoc analyses also identified that the group with MUD was less likely to repeat their first stage choices after being rewarded on the previous trial (*OR* = 0.58, *95% CI* [0.43, 0.79], *p* < 0.001), and after receiving no reward on the previous trial (*OR* = 0.70, *95% CI* [0.52, 0.95], *p* = 0.008). There was no significant difference in the combined use of the transition type and feedback from the previous trial — the behavioural proxy of model‐based decision‐making (Group * Previous Win * Previous Transition; *OR* = 1.06, *95% CI* [0.95, 1.17], *p* = 0.30). Supplementary Tables S3 and S4 include comprehensive statistics for each group when calculated separately. We note that controls did not show overly strong behavioural proxies of model‐based behaviour at baseline (Previous Win * Previous Transition; *OR* = 1.08, *95% CI* [1.00, 1.17], *p* = 0.056).

**TABLE 3:** Logistic regression predicting overt model‐based/model‐free behaviour at baseline.

Predictor	Estimate	Std error	z	*p*
Intercept	1.87	0.18	10.15	<0.001*
Previous win	0.42	0.04	10.57	<0.001*
Previous transition	−0.08	0.04	−1.89	0.059
Group	−0.89	0.26	−3.40	<0.001*
Previous win * Previous transition	0.08	0.04	1.91	0.056
Group * Previous win	−0.19	0.05	−3.62	<0.001*
Group * Previous transition	0.04	0.05	0.69	0.49
Group * Previous win * Previous transition	0.05	0.05	1.04	0.30

* *p* < 0.05.

#### 3.1.2 Computational mechanisms of model‐based/model‐free decision‐making

**FIGURE 4:**
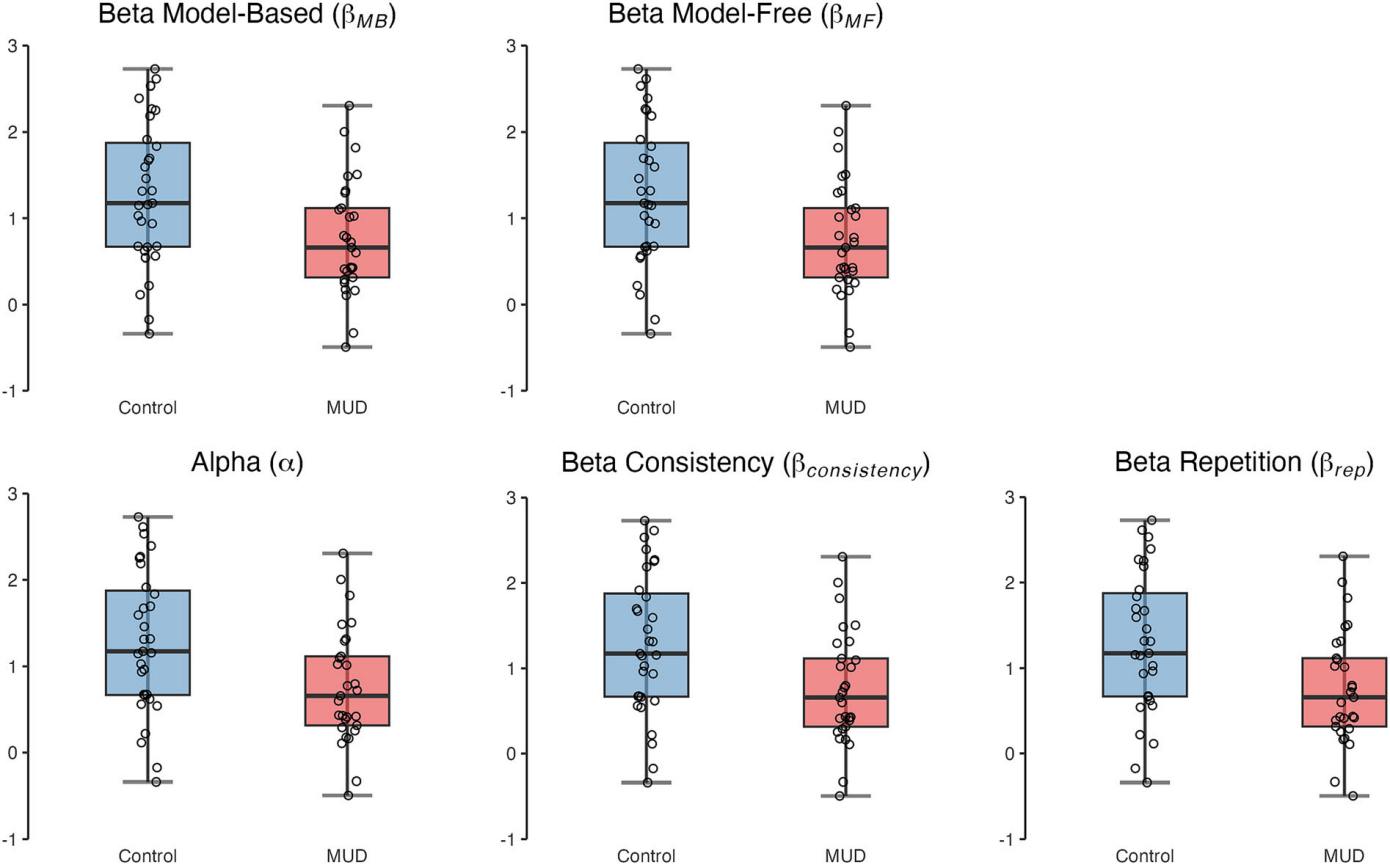
Estimated values for each parameter separated by group. *Note*: Dots represent individual participants, boxplot represents interquartile range (Q1–Q3), error bars represent to minimum/maximum. Any dots beyond are minor outliers (1.5 multiplied by IQR). For specific descriptions of each parameter, see Table 2

Consistent with the behavioural data, there was weak evidence of a null group difference in the model‐based parameter (*β*_*MB*_, *BF*_*10*_ = 0.57). There was moderate evidence to support group differences in the model‐free parameter (*β*_*MF*_; *BF*_*10*_ = 3.53), whereby people with MUD showed lower model‐free use compared with matched controls. There was weak evidence that controls learned/updated more rapidly compared with people with MUD (*α*, *BF*_*10*_ = 1.70), and moderate evidence that people with MUD showed a more consistent pattern of choosing the most subjectively rewarding stage 2 choice (*β*_*consistency*_, *BF*_*10*_ = 3.11). Finally, there was moderate evidence that matched controls were more likely to repeat previously rewarded actions, (*β*_*rep*_, *BF*_*10*_ = 3.25). Figure 4 presents each parameters' value between groups.

### 3.2 Aim 2: relationship between computational mechanisms of model‐based/model‐free decisions and methamphetamine use patterns

There was a positive relationship between recent frequency of methamphetamine use consumption and consistency of choice at stage 2 (*β*_*rep*_; *ρ* = 0.39, *p* = 0.047). The regression model predicting frequency of methamphetamine use was significant (*F*[6, 19] = 2.94, *p* = 0.03, R^2^ = 0.48). However, there was no evidence for any predictors significantly explaining variance in frequency of use. We report the full regression analyses in the Supplementary Results.

### 3.3 Aim 3: longitudinal analyses of changes in MBMF decision‐making

#### 3.3.1 Overt behaviour (stay/switch after reward and transition) over time

**FIGURE 5:**
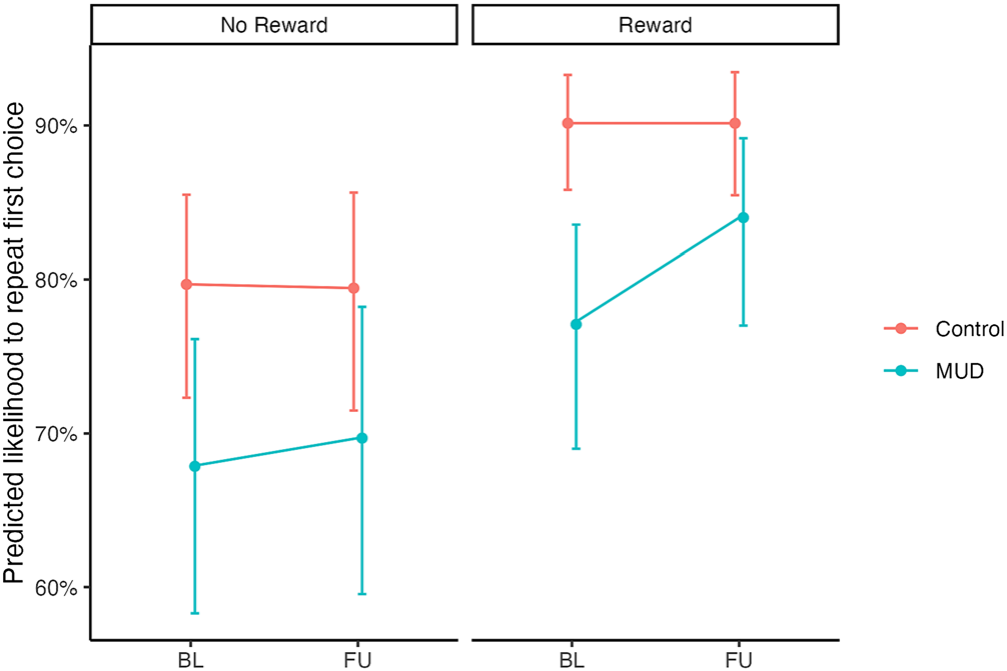
Predicted likelihood to repeat choice after different feedback across sessions. *Note*: This figure highlights how the group with MUD begin to substantially improve their repetition of rewarded actions between baseline and follow‐up

The group with MUD showed a significant improvement in their behavioural proxy of model‐free decision‐making across time compared with controls (Time * Group * Previous Win; *OR* = 1.19, *95% CI* [1.02, 1.39], *p* = 0.029). Furthermore, post‐hoc analyses found that participants with MUD significantly improved their repetition after reward (*OR* = 1.25, *95% CI* [1.03, 1.51], *p* = 0.009), though the group with MUD still had marginal differences in this behaviour compared with controls at follow‐up (*OR* = 0.76, *95% CI* [0.55, 1.04], *p* = 0.049; Figure 5. In comparison, participants with MUD did not change in their switching after non‐reward (*OR* = 1.04, *95% CI* [0.87, 1.26], *p* = 0.60). Groups did not significantly differ in the improvement of their behavioural proxies of model‐based decision‐making (Time * Group * Previous Win * Previous Transition; *OR* = 0.94, *95% CI* [0.81, 1.10], *p* = 0.44; Table 4). Supplementary Tables S8 and S9 include comprehensive statistics for each group.

**TABLE 4:** Logistic regression predicting likelihood of participants to repeat their first choice across the two timepoints (initial assessment and six‐week follow‐up).

Predictor	Estimate	Std error	z	*p*
Intercept	1.79	0.18	9.99	<0.001*
Previous Win	0.42	0.04	10.64	<0.001*
Previous Transition	−0.08	0.04	−1.89	0.058
Previous Win * Previous Transition	0.08	0.04	1.90	0.057
Group	−0.81	0.25	−3.19	0.001*
Time	−0.05	0.16	−0.05	0.96
Group * Time	0.27	0.23	1.20	0.23
Group * Previous Win	−0.19	0.05	−3.67	<0.001*
Group * Previous Transition	0.04	0.05	0.69	0.49
Group * Previous Win * Previous Transition	0.05	0.05	1.04	0.30
Time * Previous Win	0.01	0.06	0.12	0.90
Time * Previous Transition	0.08	0.06	1.28	0.20
Time * Previous win * Previous Transition	0.12	0.06	1.94	0.052
Time * Group * Previous Win	0.17	0.08	2.18	0.029*
Time * Group * Previous Transition	0.04	0.63	0.48	0.63
Time * Group * Previous Win * Previous Transition	−0.06	0.08	−0.77	0.44

#### 3.3.2 Computational mechanisms underlying overt behavioural over time

There was no evidence for changes in the model‐free parameter over time as a function of group [*β*_*MF*_; *F*(1, 45) = 0.18, *p* = 0.68, *BF*_*incl*_ = 0.18]. There was anecdotal evidence that groups did not differ in how their model‐based parameter changed over time [*β*_*MB*_; *F*(1, 45) = 1.47, *p* = 0.23, *BF*_*incl*_ = 0.57]. There was also anecdotal evidence that both groups increased their use of model‐based decision‐making at follow‐up assessment, *F*(1, 45) = 4.50, *p* = 0.04, *BF*_*incl*_ = 1.38]. There were no group differences for changes in the learning/forgetting rate [*α*; *F*(1,45) = 1.36, *p* = 0.25, *BF*_*incl*_ = 0.25], repetition bias [*β*_*rep*_; *F*(1,45) = 0.44, *p* = 0.51, *BF*_*incl*_ = 0.20] and stage two consistency [*β*_*consistently*_; *F*(1,45) = 0.66, *p* = 0.42 *BF*_*incl*_ = 0.20] parameters.

## 4 DISCUSSION

We found that 1) participants with MUD showed weakened model‐free choices, 2) increased switching after reward improved during early recovery and 3) there was no evidence that people with MUD differed from controls in model‐based decision‐making. These findings suggest that MUD‐related deficits in goal‐directed behaviour are likely driven by difficulties in learning the value of actions, rather than a dominant habitual system.

As indicated by both behavioural and computational analyses, participants with MUD made less use of the previous trial's outcome to inform the next trial's first stage choice. However, we reason that people with MUD may have more generalised difficulties in learning from previous trial outcomes, rather than having a specific deficit in the mechanism developing habitual/model‐free behaviour. This argument was supported by our behavioural and computational data showing that participants with MUD were less likely to repeat rewarded first stage choices (i.e., reduced win/stay behaviour) — indicating an underlying difficulty in learning from positive outcomes. Indeed, reduced win/stay behaviour has also been identified using other tasks of decision‐making in MUD.^25^, ^44^, ^45^ This is further supported by the group differences in alpha and beta consistency values, which aligns with previous computational studies that have observed MUD‐related deficits in updating outcome values,^46^ as well as consistently selecting high reward actions.^45^, ^47^

The behavioural analysis showed that increased switching after reward improved over time in the group with MUD. While earlier studies have identified that decision‐making can improve over time during early recovery from MUD,^22^, ^48^ ours is the first to provide a specific behavioural outcome that can explain why this is occurring (i.e., greater use of rewarding feedback to guide next choice). This finding has clinical significance as it highlights that while learning adaptive reward‐related responses can be challenging for people with MUD (e.g., attending therapy, forming new social relationships), this deficit can improve. This finding also indicates the importance of incorporating therapeutic approaches that can treat deficits in action‐outcome learning processes during the first vulnerable months of early recovery.^49^

The lack of differences in model‐based decisions between the MUD and control groups contrasts with some (but not all)^17^, ^46^ previous findings in MUD.^17^ One explanation of this discrepancy is that earlier research had controls with higher intelligence and recruited from different geographical locations. In comparison, we closely matched groups for age, education, verbal IQ and socioeconomic status. This is important because model‐based decision‐making varies across age,^50^, ^51^ is greater in people with higher intelligence^52^ and impacted by psychosocial factors.^53^ The fact that our control group, who were matched in several socioeconomic factors to the group with MUD, exhibited only modest behavioural proxies of model‐based decision‐making at baseline appears to be further evidence of this.

Our findings should be considered in the context of limitations. Our version of the Two‐Step Task does not provide incentives for model‐based decision‐making, which may have led to underutilization of this system in both groups. This might have contributed to the null group differences in model‐based computational mechanisms, as there was limited evidence of model‐based decision‐making in the overt behaviour analysis. Future work might rectify this challenge by employing a two‐stage task that encourages model‐based decision‐making^32^. Our task also did not allow investigation of whether model‐based and model‐free systems were acting complementarily, or if model‐based learning was not only acting prospectively but also to retrospectively inform the reward value of uncertain options, as suggested by recent computational neuroscience work.^54^ Furthermore, model‐based deficits and model‐free dominance might only occur in MUD for methamphetamine‐related stimuli. As such, our fictive in‐game rewards may not have sufficiently engaged this dysfunctional scenario. Finally, our participants with MUD varied in duration of abstinence at the initial assessment. Future studies may obtain greater specificity of model‐based/model‐free trajectories by recruiting participants at more confined timepoints.

## AUTHOR CONTRIBUTIONS

AHR and JM are joint first authors. AHR and AVG designed the study. AHR conducted recruitment, ran analyses, and interpreted results. JM conducted the computational modelling and analyses, and interpreted results. TT‐JC aided with analyses and interpretation of results. AHR led the manuscript writing process with JM, TT‐JC and AVG providing significant feedback and revisions. All authors approved this final version for publication.

## CONFLICT OF INTEREST STATEMENT

The authors declare no conflicts of interest.

## Supporting information


**Table S1.** Self‐reported prescribed medication, and substance use in participants with MUD.
**Table S2.** Sociodemographic group comparisons in people with MUD and drug‐free controls who attended both sessions.
**Figure S1.** Correlations between simulated and predicted parameters in a simulated dataset of 60 participants (S1).
**Table S3.** Logistic regression predicting overt model‐based (Previous win * Previous transition) and model‐free behaviour in controls at baseline.
**Table S4.** Logistic regression predicting overt model‐based (Previous win * Previous transition) and model‐free (Previous win) behaviour in MUD at baseline.
**Figure S2.** Correlation matrix of baseline computational parameters in participants with MUD and clinical indices of MUD. Spearman correlations revealed potential relationships between methamphetamine use patterns and the computational parameters.
**Table S5.** Variance Inflation Factor (VIF) estimates for each parameter. These analyses indicated low risk of multicollinearity in the parameter estimates (all VIFs < 3).
**Table S6** – Predicting baseline methamphetamine use variables using baseline computational parameters and days since last use.
**Table S7.** Longitudinal analysis of model‐based/model‐free across time in controls, used to assess test–retest reliability of mixed logistic regression approach.
**Table S8.** Logistic regression predicting overt model‐based/model‐free behaviour in controls at follow‐up.
**Table S9.** Logistic regression predicting overt model‐based/model‐free behaviour in the group with MUD at follow‐up.

## Data Availability

The data that support the findings of this study are available on request from the corresponding author. The data are not publicly available due to privacy or ethical restrictions.
