# Supplementary material for: Model‐based and model‐free mechanisms in methamphetamine use disorder
Source: Addict Biol. 2023 Dec 21;29(1):e13356. doi: 10.1111/adb.13356 (PMC10898847; doi:10.1111/adb.13356)
Supplement: Supplementary file 1 — Table S1. Self‐reported prescribed medication, and substance use in participants with MUD. Table S2. Sociodemographic group comparisons in people with MUD and drug‐free controls who attended both sessions. Figure S1. Correlations between simulated and predicted parameters in a simulated dataset of 60 participants (S1). Table S3. Logistic regression predicting overt model‐based (Previous win * Previous transition) and model‐free behaviour in controls at baseline. Table S4. Logistic regression predicting overt model‐based (Previous win * Previous transition) and model‐free (Previous win) behaviour in MUD at baseline. Figure S2. Correlation matrix of baseline computational parameters in participants with MUD and clinical indices of MUD. Spearman correlations revealed potential relationships between methamphetamine use patterns and the computational parameters. Table S5. Variance Inflation Factor (VIF) estimates for each parameter. These analyses indicated low risk of multicollinearity in the parameter estimates (all VIFs < 3). Table S6 – Predicting baseline methamphetamine use variables using baseline computational parameters and days since last use. Table S7. Longitudinal analysis of model‐based/model‐free across time in controls, used to assess test–retest reliability of mixed logistic regression approach. Table S8. Logistic regression predicting overt model‐based/model‐free behaviour in controls at follow‐up. Table S9. Logistic regression predicting overt model‐based/model‐free behaviour in the group with MUD at follow‐up. [file ADB-29-e13356-s001.docx]

**Model-based and Model-Free Mechanisms in Methamphetamine Use Disorder**

Alex H. Robinson,^a^ Justin Mahlberg,^a^ Trevor T.-J. Chong,^a^ and Antonio Verdejo-Garcia^a*^

**Supplementary Materials**

^a^ Turner Institute for Brain and Mental Health, School of Psychological Sciences, Monash University, Melbourne, Australia

# Supplementary Methods

**Table S1**. Self-reported prescribed medication, and substance use in participants with MUD.

|  | | | | *N (or M)* | | % (or *SD*) | | |
| --- | --- | --- | --- | --- | --- | --- | --- | --- |
|  | *Other Illicit Subs.Use* |  | | | | |  |  |
| Cannabis | | | 10 | | 33.33 | | |  |
| GHB | | | 7 | | 23.33 | | |  |
| MDMA | | | 5 | | 16.66 | | |  |
| Cocaine | | | 3 | | 10 | | |  |
| Heroin | | | 1 | | 3.33 | | |  |
| SDS Alcohol | | | 1.13 | | 2.30 | | |  |
| SDS Cannabis | | | 1.5 | | 3.07 | | |  |
| *Prescribed Medication* | | |  | |  | | |  |
| Anti-Dep | | | 7 | | 23.33 | | |  |
| Z-Drug (Zopiclone) | | | 1 | | 3.33 | | |  |

*Note*: Other Illicit Substance Use refers to substances taken more than 10 times in the past 12 months. SDS: Severity of Dependence Scale, scores can range between 0 and 15. Anti-Dep includes escitalopram, fluoxetine, mirtazapine, venlafaxine, duloxetine.

**Table S2**. Sociodemographic group comparisons in people with MUD and drug-free controls who attended both sessions.

| Demographics | PwMUD | HC | Test Statistic | Bayes Factor |
| --- | --- | --- | --- | --- |
| Sex (F/M) | 5/17 | 7/19 | *ꭓ*^2^ = 0.11, *p* = .74 | 0.32 |
| Age | 35.05 (10.00) | 31.92 (9.05) | *U* = 237.5, *p* = .32 | 0.47 |
| Years of Education | 14.14 (2.41) | 14.58 (1.91) | *U* = 297, *p* = .83 | 0.33 |
| Verbal IQ | 111.20 (5.24) | 111.78 (4.47) | *U* = 302.5, *p* = .74 | 0.30 |
| Sociodem. Status | 6.64 (2.54) | 7.04 (2.39) | *U* = 298, *p* = .63 | 0.33 |

*Note*: Three PwMUD also reported HIV+ status.

**Supplementary Results**

**Figure S1.** Correlations between simulated and predicted parameters in a simulated dataset of 60 participants (S1).


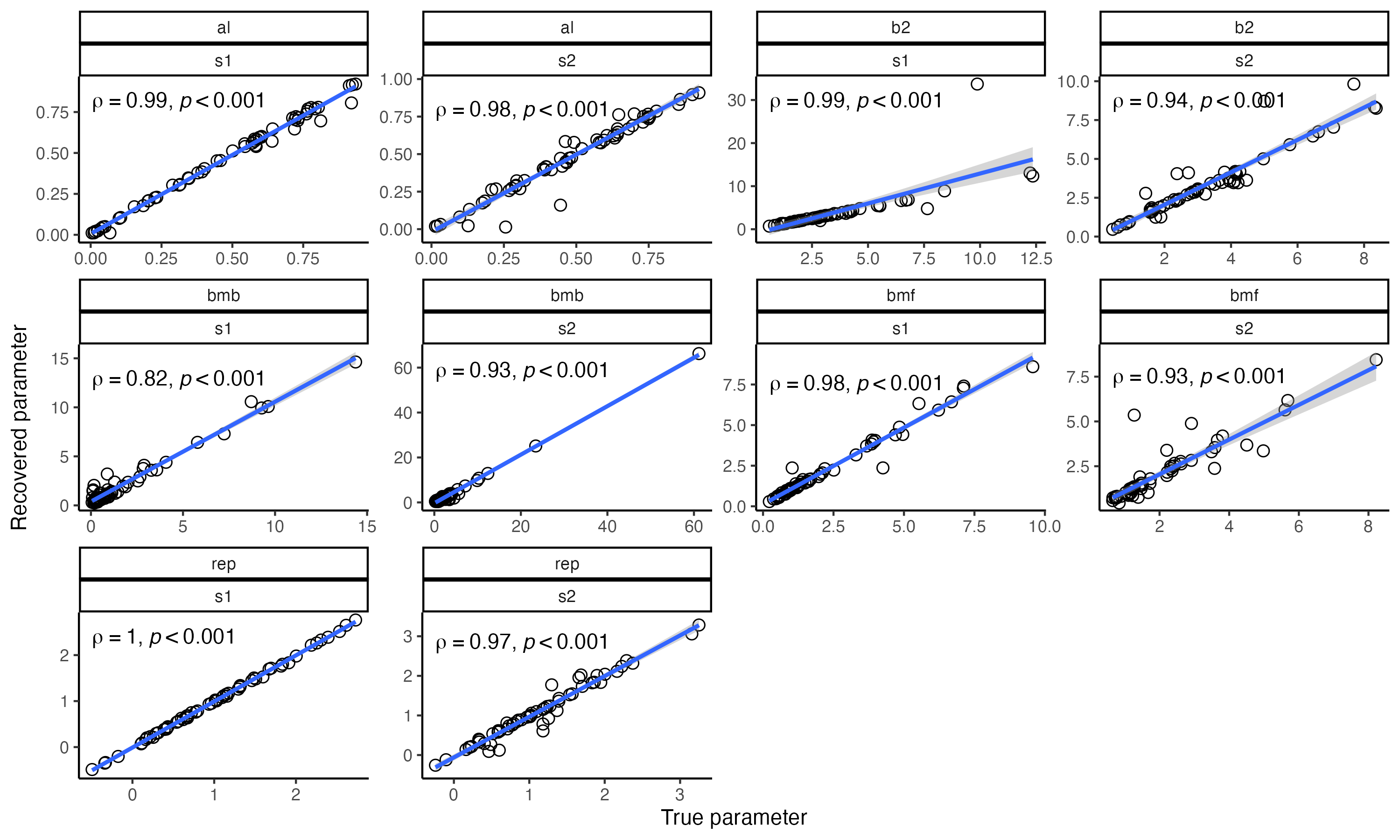


## Aim 1: Baseline Differences Between Groups

**Table S3.** Logistic regression predicting overt model-based (Previous win * Previous transition) and model-free behaviour in controls at baseline.

| **Predictor** | **Estimate** | **Std Error** | **z** | ***p*** |
| --- | --- | --- | --- | --- |
| Intercept | 1.89 | 0.21 | 8.80 | <.001* |
| Previous win | 0.42 | 0.04 | 10.59 | <.001* |
| Previous transition | - 0.08 | 0.04 | -1.90 | .059 |
| Previous win * Previous transition | 0.08 | 0.04 | 1.91 | .056 |

**Table S4.** Logistic regression predicting overt model-based (Previous win * Previous transition) and model-free (Previous win) behaviour in MUD at baseline.

| **Predictor** | **Estimate** | **Std Error** | **z** | ***p*** |
| --- | --- | --- | --- | --- |
| Intercept | 0.97 | 0.15 | 6.40 | <.001* |
| Previous win | 0.23 | 0.03 | 6.93 | <.001* |
| Previous transition | - 0.04 | 0.03 | -1.17 | .24 |
| Previous win * Previous transition | 0.13 | 0.03 | 3.88 | <.001* |

## Aim 2: Relationships Between Decision-Making and Methamphetamine Use Patterns

**Figure S2.** Correlation matrix of baseline computational parameters in participants with MUD and clinical indices of MUD. Spearman correlations revealed potential relationships between methamphetamine use patterns and the computational parameters.
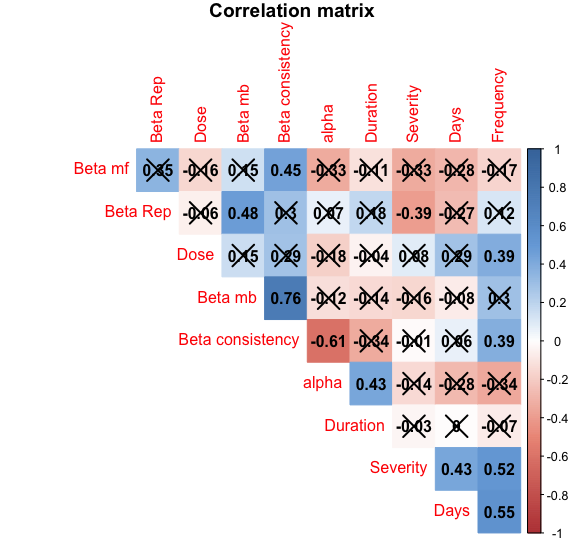


*Note*: *Crosses indicate non-significant correlations (p > .05).*

**Table S5. Variance Inflation Factor (VIF) estimates for each parameter.** These analyses indicated low risk of multicollinearity in the parameter estimates (all VIFs < 3).

| Variance inflation factors for predictors in each regression model | | | | | | | |
| --- | --- | --- | --- | --- | --- | --- | --- |
|  | Outcome | Alpha (α) | Beta Rep (*βrep*) | Beta Consistency  (*β_consistency_*) | Beta MB (*β_MB_*) | Beta MF (*β_MF_*) | Days |
|  | | | | | | | |
| 1 | Severity | 1.79 | 1.56 | 2.66 | 2.31 | 1.35 | 1.41 |
| 2 | Days | 1.75 | 1.56 | 2.40 | 2.15 | 1.16 | NA |
| 3 | Dose | 1.73 | 1.55 | 2.73 | 2.35 | 1.29 | 1.43 |
| 4 | Duration | 1.79 | 1.56 | 2.66 | 2.31 | 1.35 | 1.41 |
| 5 | Frequency | 1.79 | 1.55 | 2.66 | 2.31 | 1.35 | 1.41 |
| *Note:* Severity = Severity of Dependence Scale; Days = Days since they last used methamphetamines; Dose = the average dosage of methamphetamines used; Duration = Duration of Methamphetamine use Disorder; Frequency = number of days where methamphetamines (timeline follow back). Variance inflation factor estimates > 10 are considered indicative of significant multicollinearity issues. | | | | | | | |

**Table S6 – Predicting baseline methamphetamine use variables using baseline computational parameters and days since last use.**

|  | **Severity** | | | **Days** | | | **Dose** | | | **Duration** | | | **Frequency** | | |  |
| --- | --- | --- | --- | --- | --- | --- | --- | --- | --- | --- | --- | --- | --- | --- | --- | --- |
| *Predictors* | *B* | *SE* | *p* | *B* | *SE* | *p* | *B* | *SE* | *p* | *B* | *SE* | *p* | *B* | *SE* | *p* |  |
| (Intercept) | 1.82 | 3.52 | 0.611 | 6.27 | 18.56 | 0.739 | 0.23 | 0.20 | 0.267 | -5.89 | 3.99 | 0.156 | 6.15 | 7.02 | 0.392 | |
| Alpha (α) | -0.70 | 3.92 | 0.861 | -13.63 | 20.50 | 0.514 | -0.34 | 0.22 | 0.145 | 8.51 | 4.44 | 0.071 | -11.41 | 7.82 | 0.161 | |
| Beta Consistency  (*β_consistency_*) | -1.12 | 1.58 | 0.486 | 0.51 | 8.34 | 0.951 | -0.10 | 0.09 | 0.259 | -0.17 | 1.79 | 0.926 | 3.47 | 3.15 | 0.284 | |
| Beta Rep (*βrep*) | 0.13 | 0.64 | 0.841 | 4.80 | 3.21 | 0.150 | -0.02 | 0.04 | 0.566 | -0.67 | 0.73 | 0.368 | -0.65 | 1.28 | 0.619 | |
| Beta MB (*β_MB_*) | -0.19 | 0.55 | 0.734 | -3.38 | 2.79 | 0.240 | 0.06 | 0.03 | 0.085 | 0.53 | 0.62 | 0.408 | 1.14 | 1.09 | 0.310 | |
| Beta MF (*β_MF_*) | -0.41 | 0.47 | 0.394 | -4.13 | 2.31 | 0.089 | -0.02 | 0.03 | 0.443 | 0.70 | 0.53 | 0.206 | -1.44 | 0.94 | 0.141 | |
| Days | 0.04 | 0.04 | 0.387 |  |  |  | 0.00 | 0.00 | 0.987 | 0.07 | 0.05 | 0.149 | 0.16 | 0.08 | 0.069 | |
| Observations | 26 | | | 26 | | | 24 | | | 26 | | | 26 | | |  |
| R^2^ | 0.194 | | | 0.293 | | | 0.316 | | | 0.323 | | | 0.481 | | |  |
| F | 0.76 | | | 1.66 | | | 1.31 | | | 1.51 | | | 2.94* | | |  |
| *Note:* Severity = Severity of Dependence Scale; Days = Days since they last used methamphetamines; Dose = the average dosage of methamphetamines used; Duration = Duration of Methamphetamine use Disorder; Frequency = number of days where methamphetamines (timeline follow back). *B* = regression coefficient estimate; *SE =* standard error of the estimate; *p* = significance test value. R^2^ = Multiple R squared, i.e. variance in the outcome measure that is explained by the predictors in the model. A*sterisks indicate p < .05* | | | | | | | | | | | | | | | |  |

## Aim 3: Longitudinal Analyses of Changes in Model-based/Model-free Decision-Making

### ***Sensitivity Analyses of Test-Retest Reliability***

To assess test-retest stability in the overt behaviour analyses (i.e., mixed logistic regression), we examined whether the control group had any significant interactions between Time and any fixed effects. We chose to only investigate the control group in this analysis as they were not engaged in any treatment/recovery processes and thus should have more stable decision-making across time. This approach did not find any significant interactions (Table S7) and thus it appeared that these measures remained relatively stable across time.

We calculated test-retest reliability of the computational parameters using the interclass coefficient (ICC). Here, the reliability indexes were poor across all variables other than beta consistency (*α* = .41, *β_consistency_* = .81, *β_MB_* = .43, *β_MF_* = .37, *β_rep_* = .59).^2^ However, it can be expected that within-individual variations can occur when recovering multiple, related parameters of computational modelling.^3^ Furthermore, another study using the same model^4^ found more acceptable test-retest reliabilities using a more sophisticated reliability estimates approach that accounted for different trajectories amongst individuals.^5^

**Table S7.** Longitudinal analysis of model-based/model-free across time in controls, used to assess test-retest reliability of mixed logistic regression approach.

| **Predictor** | **Estimate** | **Std Error** | **z** | ***p*** |
| --- | --- | --- | --- | --- |
| Intercept | 1.81 | 0.21 | 8.73 | <.001* |
| Previous win | 0.43 | 0.04 | 10.66 | <.001* |
| Previous transition | - 0.08 | 0.04 | -1.89 | .059 |
| Previous win * Previous transition | 0.08 | 0.04 | 1.90 | .058 |
| Time | -0.01 | 0.19 | -0.07 | .94 |
| Time * Previous win | 0.01 | 0.06 | 0.10 | .92 |
| Time * Previous transition | 0.08 | 0.06 | 1.27 | .20 |
| Time * Previous win * Previous transition | 0.12 | 0.06 | 1.94 | .052 |

**Table S8.** Logistic regression predicting overt model-based/model-free behaviour in controls at follow-up.

| **Predictor** | **Estimate** | **Std Error** | **z** | ***p*** |
| --- | --- | --- | --- | --- |
| Intercept | 1.87 | 0.21 | 8.77 | <.001* |
| Previous win | 0.43 | 0.04 | 9.77 | <.001* |
| Previous transition | 0.00 | 0.04 | -0.01 | .99 |
| Previous win * Previous transition | 0.19 | 0.04 | 4.33 | <.001* |

**Table S9.** Logistic regression predicting overt model-based/model-free behaviour in MUD group at follow-up.

| **Predictor** | **Estimate** | **Std Error** | **z** | ***p*** |
| --- | --- | --- | --- | --- |
| Intercept | 1.34 | 0.21 | 6.47 | <.001* |
| Previous win | 0.41 | 0.04 | 10.24 | <.001* |
| Previous transition | 0.07 | 0.04 | 1.83 | .067 |
| Previous win * Previous transition | 0.18 | 0.04 | 4.59 | <.001* |

References

1. Thompson CG, Kim RS, Aloe AM, Becker BJ. Extracting the variance inflation factor and other multicollinearity diagnostics from typical regression results. *Basic Appl Soc Psychol*. 2017;39(2):81–90. doi:10.1080/01973533.2016.1277529

2. Koo TK, Li MY. A guideline of selecting and reporting intraclass correlation coefficients for reliability research*. J Chiropr Med*. 2016;15(2):155–63. doi:10.1016/j.jcm.2016.02.012

3. Brown VM, Chen J, Gillan CM, Price RB. Improving the reliability of computational analyses: Model-based planning and its relationship with compulsivity. *Biol Psychiatry Cogn Neurosci Neuroimaging*. 2020;5(6):601–609. doi:10.1016/j.bpsc.2019.12.019

4. Foerde K, Daw ND, Rufin T, Walsh BT, Shohamy D, Steinglass JE. Deficient goal-directed control in a population characterized by extreme goal pursuit. *J Cogn Neurosci*. 2021;33(3):463–481. doi:10.1162/jocn_a_01655

5. Haaf JM, Rouder JN. Some do and some don’t? Accounting for variability of individual difference structures. *Psychon Bull Rev*. 2019;26(3):772–789. doi:10.3758/s13423-018-1522-x
